# Uncertain Water Environment Carrying Capacity Simulation Based on the Monte Carlo Method–System Dynamics Model: A Case Study of Fushun City

**DOI:** 10.3390/ijerph17165860

**Published:** 2020-08-13

**Authors:** Xian’En Wang, Wei Zhan, Shuo Wang

**Affiliations:** 1 School of New Energy and Environment, Jilin University, Changchun 130021, China; wxen@jlu.edu.cn (X.E.); zhanwei18@mails.jlu.edu.cn (W.Z.); 2 Key Laboratory of Groundwater Resources and Environment, Jilin University, Ministry of Education, Changchun 130021, China; 3 Jilin Provincial Key Laboratory of Water Resources and Environment, Jilin University, Changchun 130021, China

**Keywords:** water environment carrying capacity, uncertainty, Monte Carlo method, system dynamics

## Abstract

Water environment carrying capacity (WECC) is an effective indicator that can help resolve the contradiction between social and economic development and water environment pollution. Considering the complexity of the water environment and socioeconomic systems in Northeast China, this study establishes an evaluation index system and a system dynamics (SD) model of WECC in Fushun City, Liaoning, China, through the combination of the fuzzy analytic hierarchy process and SD. In consideration of the uncertainty of the future development of society, the Monte Carlo and scenario analysis methods are used to simulate the WECC of Fushun City. Results show that if the current social development mode is maintained, then the WECC in Fushun will have a slow improvement in the future, and a “general” carrying state with a WECC index of 0.566 in 2025 will be developed. Moreover, focusing on economic development (Scheme 1 with a WECC index of [0.22, 0.45] in 2025) or environmental protection (Scheme 2 with a WECC index of [0.48, 0.68] in 2025) cannot effectively improve the local water environment. Only by combining the two coordinated development modes (Scheme 3) can WECC be significantly improved and achieve “general” or “good” carrying state with a WECC index of [0.59, 0.79]. An important development of this study is that the probability of each scheme’s realization can be calculated after different schemes are formulated. In turn, the feasibility of the scheme will be evaluated after knowing the probability, so as to determine the path suitable for local development. This is of great significance for future urban planning.

## 1. Introduction

The frequency of water shortages and environmental pollution caused by population growth and rapid economic growth has been increasing since the 1980s [1,2]. Water environment carrying capacity (WECC) is defined as the largest population and economic scale that the water environment can support in a specific region for some period without evident adverse effects on the local water environment [3]. WECC has been studied for human–water environment sustainability [4].

Since the 1990s, researchers have been working on the relationship between the water environment and social economy [5]. For example, Wei et al. (2014) developed an index system that can be used to assess land carrying capacity through the conceptual model of “driving force–pressure–state–response–control” [6]. He used the index system method, which is a static evaluation method, and it is difficult to reflect the nonlinear process and the interaction between elements in environmental science. Therefore, it is rarely used alone, and is generally used in combination with other methods. For example, Qian et al. (2015) conducted a comparative analysis of the land carrying capacity of Xiamen city based on ecological footprint analysis and the indicator system method [7]. The ecological footprint method is also a static evaluation method and lacks analysis of the interaction mechanism between system elements. In order to study the system more effectively, the system dynamics (SD) method is applied. For example, Yang et al. (2015) applied the coupling system dynamics method to assess the water environmental carrying capacity for sustainable development in Tieling, Liaohe [8]. This method is a dynamic method, which can establish the coupling relation of various system elements and simulate and predict the bearing condition of the regional composite system with the help of the input and output of the model. However, there are still some deficiencies in forecasting, so how to make up for them is the focus of this paper.

From the author’s point of view, WECC system in the real world is complex [9], and its operation and development are accompanied by a large number of uncertainties. Strictly speaking, it is impossible to be absolutely certain of the value of some element in the system at some point in the future, even if there is a 99% certainty, there is a 1% uncertainty. Therefore, it is more reasonable to give an interval where the values can vary. The probability of each value in this interval is different, and our research is to find this interval and the probability of each value in this interval. Such a prediction of the system’s development would be convincing. Moreover, from the previous studies of WECC, the lack of uncertainty consideration and uncertainty analysis had produced undesirable consequences. For example, the distortion of the evaluation results would lead to the failure of the program based on the evaluation results, and the research results may not provide an effective basis for policy making. Therefore, it is necessary to analyze the uncertainty of general WECC evaluation results with effective methods, both theoretically and practically.

Considering the advantage of system dynamics, the Monte Carlo method is introduced and coupled in this study, Taking Fushun city as an example, and this paper discusses the problem of WECC uncertainty prediction and puts forward some measures for the current poor water environment of Fushun City.

## 2. Materials and Methods

### 2.1. Study Area and Data

**Figure 1:**
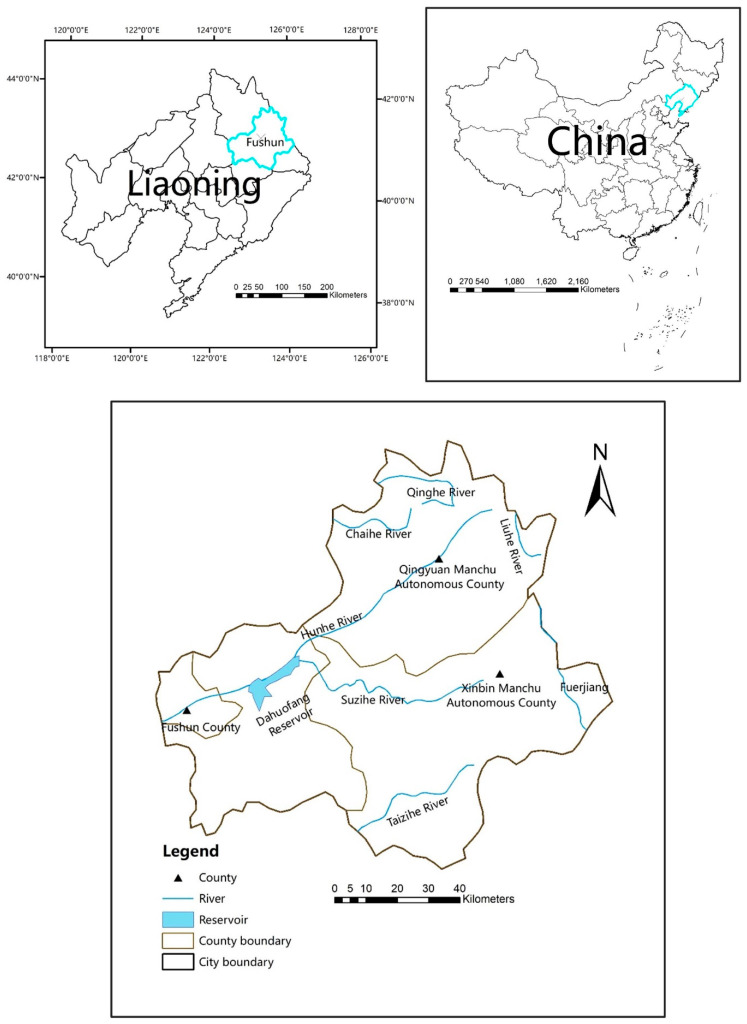
Location of the study area and distribution of major rivers in the area.

Fushun (123°39′–125°28′ E, 41°14′–42°28′ N), which is located east of Liaoning Province (Figure 1), covers an area of 11,271.03 square kilometers. Fushun is an important water source protection area of Liaoning Province, an important industrial base, and a subcentral city of the Shenyang Economic Zone. The total population of the city was 2.158 million in 2015, and the GDP was recorded at RMB 121.65 billion. The average precipitation of the city was 690.3 mm, and the water resources totaled 1.951 billion m3. Fushun is rich in water resources, but the ecological functions of rivers and other water bodies declined due to an increase in industrial and agricultural water and sewage discharge.

**Figure 2:**
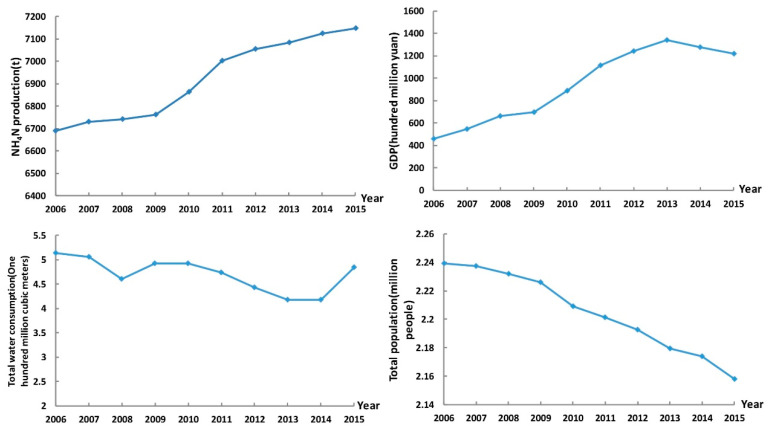
The change trend of total water consumption, NH_4_N production, total population and GDP from 2006 to 2015.

Hydrological data, such as total water consumption, is derived from the Fushun Water Resources Bulletin [10]. Pollutant related data, such as NH_4_N emissions, are gathered from the Fushun Statistical Yearbook [11]. Other socioeconomic data, such as total population and GDP, are obtained from the Fushun Yearbook [12]. Figure 2 shows the change trend of total water consumption, NH_4_N production, total population and GDP from 2006 to 2015.

### 2.2. Methodology

**Figure 3:**
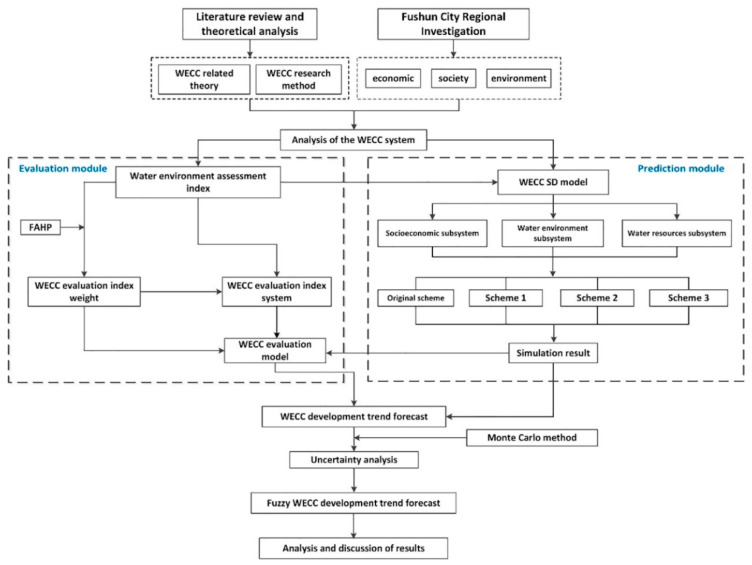
Technological route map.

The main framework of this study is shown in Figure 3, mainly using four methods and divided into two modules. Combining these two modules can obtain the future development trend of the Fushun WECC index. The Monte Carlo method is introduced to analyze the uncertainty of some water environment evaluation indicators, in this manner, the predicted values of the four schemes become more realistic and accurate. Finally, the fuzzy WECC development trend is obtained. On this basis, policy recommendations are proposed.

The first module is the evaluation module. After analyzing the WECC system of Fushun City, the water environment evaluation index is selected. Based on a fuzzy analytic hierarchy process (FAHP), the paper constructs a regional water environment carrying capacity evaluation index system to reflect the causal relationship between human social development and water environment pollution [13]. The second module is the prediction module. The WECC SD model is established after the water environment evaluation index is selected. The model is divided into three subsystems, namely the social–economic subsystem, the water environment subsystem, and the water resource subsystem. To alleviate the contradiction between economic development and the water environment in the Fushun area, four schemes were designed for comparative study, and the values of various evaluation indicators in 2016–2025 were predicted, respectively. Combined with the weight of the evaluation indicators in the evaluation module, the WECC evaluation model is obtained by using the standardization method of indicators.

Combining these two modules can get the future development trend of the Fushun WECC index. To make the predicted values of the four schemes more realistic and accurate, the Monte Carlo method is introduced to analyze the uncertainty of some water environment evaluation indicators, and finally, the fuzzy WECC development trend is obtained. Based on this, policy recommendations are proposed.

#### 2.2.1. WECC Index System and Index Weight Calculation with FAHP

The establishment of a scientific and reasonable index system would help provide a basis for the accurate assessment of the regional WECC [14]. This study selected 16 indicators to reflect the ecosystem health status of Fushun. After considering the actual situation of the study area and consulting with experts, the system was divided into three categories: water resource, water environment, and human society. In the present study, some indicators were selected to reflect the ecosystem health status using available data. Finally, after considering the actual conditions in the study area and consulting with experts, the evaluation index system for the WECC in the Fushun area was established (Table 1), and the system was classified into three categories, i.e., water resources, water environment, and human society, using 16 indicators in total.

The AHP is a decision analysis method developed by Satty [15]. This method considers qualitative and quantitative information. As a result, the AHP has a special advantage in multi-index evaluation. AHP has been applied in many research fields, including nature, economy, and society [16]. However, the construction of the pairwise comparison judgment matrix usually does not consider the ambiguity of human judgment and only considers the possible extreme situation of human judgment in the AHP of general problems [17]. When conducting expert consultation on some issues, experts often provide some amount of ambiguity (such as a three-value judgment, namely, the lowest possible value, highest possible value, and most likely value). In view of such ambiguity, Laarhoven and Pedrycz evolved AHP into FAHP, bringing the triangular fuzzy number of fuzzy set theory directly into the pairwise comparison matrix of the AHP [18]. The purpose is to solve vague problems, which occur during the analysis of criteria and the judgment process. FAHP tolerates vagueness or ambiguity and should be more appropriate and effective than conventional AHP in real practice [19]. Therefore, FAHP has been increasingly used by researchers to solve various problems in recent years. Wu et al. [20] used FAHP to evaluate the performance indicators of intellectual capital in the ecological economy industry. Sivasangari, A et al. [21] used FAHP to analyze and predict breast cancer.

In this study, trigonometric fuzzy numbers are used to improve AHP. The equations are shown in Formulas (1)–(4) in Table 2.

Finally, the relative weight of each evaluation index in the system was obtained (Table 1).

#### 2.2.2. System Dynamics Model

The SD model starts from the internal structure of the system, and the first-order differential equations are used to reflect the causal feedback relationship among the variables in the system; hence, the SD simulation model is established. The policy simulation of different development schemes is first performed in the evaluation of carrying capacity, and then the decision variables are predicted. The decision variables are taken as the index system of the carrying capacity evaluation, and then the index evaluation method is combined for calculation and analysis. Lastly, the optimal development scheme and the corresponding carrying capacity are obtained. The SD model method simulates the behavior of a real-world nonlinear complex system over time and has advantages in dynamic monitoring, which can consider the interaction between various factors within the system [22]. This method is suitable for dealing with long-term and periodic problems and is an effective tool for analyzing complex systems [23].

In this study, the administrative boundaries of Fushun City (including Xinfu District, Wanghua District, Dongzhou District, Shuncheng District, Fushun County, Xinbin Manchu Autonomous County, and Qingyuan Manchu Autonomous County) are taken as the system boundaries. The simulation time is 2016–2025, and the simulation time interval is one year. In accordance with the WECC evaluation index system (Table 1) and the social and economic development status of the Fushun area, the WECC system is divided into three subsystems: social and economic, water resource, and water environment. 

The WECC SD model as a simulation model for an actual system must be consistent with the objective reality. In this study, DP, total population, urban population, COD production, _NH4N_ production, and total water consumption were selected as the major variables for a historical test to confirm the validity of the model after the sensitivity analysis of the model parameters. Table 3 shows the calculated relative error between the simulated and actual values. The results show that the model error is less than 10%, the test error is within the allowed range, and the simulation period is very short. Therefore, the model can represent the real system and meet the requirements of prediction.

#### 2.2.3. WECC Evaluation Model

Given that WECC evaluation indicators have different dimensions, units, and sizes, evaluating them comprehensively is difficult. Therefore, this study divides the evaluation indicators into three categories, namely, “the greater, the better;” “the smaller, the better;” and “the closer a certain value is, the better”. The index standardization method is used to determine the standard score for each evaluation indicator, and the WECC index is introduced to quantitatively describe the running state of the system. The equations are shown in Formulas (5)–(8) in Table 2.

Many WECC studies are limited to qualitative descriptions, but several quantitative analyses have been performed [24]. No uniform classification and calculation method for carrying capacity has been identified. Therefore, based on the membership degree and water environment carrying capacity state classification method in fuzzy mathematics, the carrying state is divided into five groups according to the WECC index value (Table 4).

#### 2.2.4. Monte Carlo Method

The Monte Carlo method is a stochastic simulation method based on probabilistic and statistical theoretical methods. The basic idea is to link the problem that needs to be solved with a probability model and generate a series of random numbers for the model through the computer. All the statistical characteristic values of the parameters are obtained after repeated calculation, and the approximate values of the results are given. The accuracy of the result can be expressed by the standard error of the estimate. The Monte Carlo method can randomly simulate the dynamic relationship among various variables and estimate the distribution of model results through repeated simulation to solve several complex problems with uncertainty [25]. In recent years, this method has often been used in conjunction with other methods, such as with Fermi DwiWicaksono et al. [26], who coupled the FAHP and Monte Carlo methods and proposed a new multi-criteria decision analysis method based on the Normative Monte Carlo Fuzzy Analytic Hierarchy Process (NMCFAHP), which combined the fuzziness of judgment with probability uncertainty and cognitive uncertainty. This study also combines system dynamics and Monte Carlo method to study WECC in Fushun region. Below are the general steps for using Monte Carlo method.

Assume a function *Y* = *f*(*x*_1_, *x*_2_, *x*_3_, …, *x_n_*), where *x*_1_, *x*_2_, *x*_3_, …, *x_n_* is a statistically independent random variable, and the probability distribution is known. In practical problems, *f*(*x*_1_, *x*_2_, *x*_3_, …, *x_n_*) is often unknown or a complex functional relationship and has difficulty solving relevant probability distributions and numerical features using analytical methods. The Monte Carlo method uses a random number generator to extract the value *x*_1*i*_, *x*_2*i*_, *x*_3*i*_, …, *x_ni_* of each set of random variables *x*_1_, *x*_2_, *x*_3_, …, *x_n_* through direct or indirect sampling and determines the value of the function *Y* by the relation of *Y* to *x*_1_, *x*_2_, *x*_3_, …, *x_n_*:*Y_i_* = *f*(*x*_1*i*_, *x*_2*i*_, *x*_3*i*_, …, *x_ni_*).

A batch of sampled data of the function *Y* can be obtained by repeatedly sampling multiple times (*i* = 1, 2, 3, …). When the number of simulations is sufficient, the probability distribution and digital features of function *Y*, which are close to the actual situation, can be provided.

## 3. Results and Discussion

### 3.1. Results

#### 3.1.1. Analysis of the Current Situation of the Water Environment Carrying Capacity in Fushun City

The WECC index of the Fushun area in 2015 was 0.398 after calculation. Overall, WECC was in a “poor” carrying state, which indicates that the water environment in the Fushun region has been poor and polluted in recent years [27]. From a partial point of view, the author analyzed the current situation of the WECC in the Fushun area from the aspects of social economy, water resources, and water environment.

This study selected the total population, urbanization level, GDP, and the growth rate of the three industries to describe the social and economic conditions of Fushun City. The total population of Fushun City was 2.158 million in the end of 2015, of which the urban population was 1.489 million, and the rural population was 0.669 million. Compared with the data of previous years, the total population and urbanization water showed a downward trend. Population is a key constraint to economic development in the process of social development. The population quantity and quality directly determine the speed and scale of economic development; thus, the population of Fushun City reduced, especially the urban population, thus inevitably affecting economic development. Policies can be formulated to attract population, increase the total population of Fushun City, and improve the level of urbanization to promote economic development in the future. Another important indicator in the social and economic system is GDP and the growth rate of three industries. As the most direct indicator of the level of economic development, Fushun City’s GDP in 2015 was RMB 121.65 billion, a slight increase from the previous year (calculated at comparable prices). The growth rate of three industries were 4.4%, 1.6%, and 7.1%, respectively. The economic development was slow, with the second industry growth rate still falling. Investment in agriculture and industry can be increased to speed up economic development in the future.

The water resource system had the least impact on the WECC in the three systems because of many rivers in Fushun, including the Hunhe, Qinghe, Chaihe, Fuerjiang, and Liuhe rivers. Hence, the water reserves are sufficient. The total water consumption of Fushun City in 2015 was 485 million m3, including 259 million m3 for the primary industry and 158 million m3 for the secondary industry. Although water consumption has increased from last year, the total water consumption and the trend of the three-production water consumption have been declining in recent years. The ratio of water supply to consumption in Fushun in 2015 was 1.56, which clearly showed that the amount of water in Fushun can meet the needs of the entire city. Appropriate water-saving policies should be formulated in the future, but economic development should not be affected.

WECC is affected by the water environment system, and the current water environment in Fushun is not remarkable. The COD and NH_4_N emissions of Fushun City were 10,400 and 4491.39 t in 2015, respectively. Both decreased but were not evident in comparison with that from the previous year. Pollutant emission remains large, and the environmental situation is grim. Two major approaches to control pollutants in the future are identified. The first approach is reducing the production of industrial and agricultural pollutants of ten thousand Yuan of added value starting from the pollutant source. The feasibility of this method is not high because the traditional production process of industry and agriculture is difficult to change in a short time, and the effect of several small improvements is limited. The second approach involves strengthening the research and development of pollutant treatment technology, improving the treatment rate of pollutants, and reducing the emission of pollutants from the treatment of pollutants.

In accordance with the present situation of water environment in Fushun, two major methods were identified to improve the WECC. First, pollutant emissions must be controlled. Second, the speed of economic development must be accelerated. Table 1 shows that the indices with great impact on the WECC of Fushun at present are COD emission, NH_4_N emission, total population urbanization rate, and GDP. This study aims to improve Fushun’s WECC by controlling these indicators.

#### 3.1.2. Scenario Analysis

In order to explore a suitable road for the sustainable development of the Fushun area in the future, this study proposes four development schemes for comparative study (as shown in Table 5), and the important evaluation indicators in different schemes need to select some key indicators to control. Total population and urbanization rate are important evaluation indices of social development, among which the total population is mainly affected by birth rate, death rate, immigration rate, and emigration rate. Due to the difficulty of human intervention in birth rate and death rate, this study selected the coefficient of migration and the coefficient of migration as the key parameters to control the total population, and selected the urbanization rate as the key parameter to balance the urban population and the rural population. GDP is the most direct evaluation index of economic development, and the growth rate of the three industries has an impact on it. Therefore, the growth rate of the three industries (calculated at comparable prices) is chosen as the key parameter to control GDP. The main pollutants discharged into the water environment in Fushun area are COD and NH_4_N, so the COD treatment rate and NH_4_N treatment rate are taken as the key parameters to control the discharge of COD and NH_4_N in this study. This study mainly controls the development of the scheme by controlling the value of key parameters.

**Figure 4:**
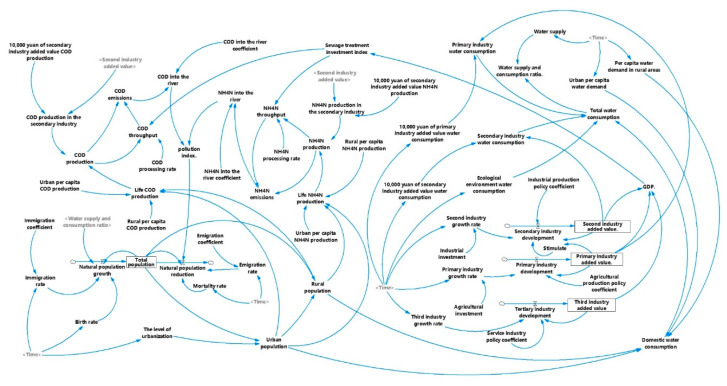
System dynamics flow chart of WECC in Fushun.

In the present study, Vensim Software is used to establish the SD model of WECC for Fushun and to perform the simulation. The SD flowchart of the WECC system is shown in Figure 4. This study took 2015 as the base year and implemented the scheme from the beginning of 2016. The SD model is used to simulate the numerical changes of Fushun’s social and economic development, water resource utilization, and pollutant emission under the background of four development schemes from 2016 to 2025. Lastly, the WECC index was calculated to evaluate WECC, focusing on the development results of the WECC index of four schemes.

**Figure 5:**
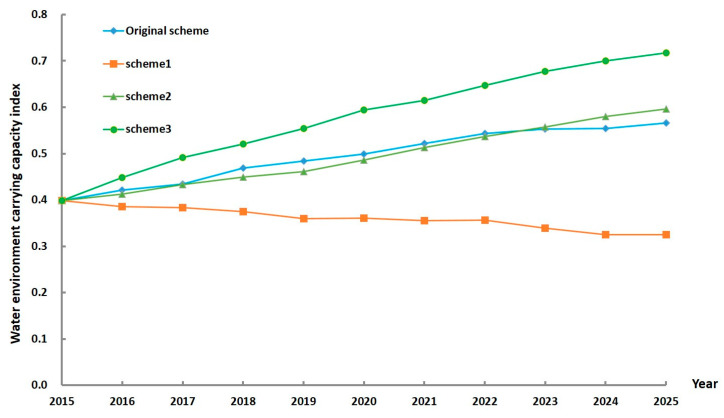
Results of WECC index in different schemes.

As shown in Figure 5, except for the slow decline of WECC in Scheme 1, the future development of WECC in the other three schemes is on the rise. In 2015, the WECC index was 0.398. In the original scheme, the WECC rises slowly and can only achieve a “general” carrying state with a WECC index of 0.566 in 2025. The WECC trend in Scheme 2 is very similar to the original one. Although the WECC index can reach 0.597 in 2025, which is slightly higher than the original scheme, the WECC index in 2016–2022 is lower than the original scheme, so the environmental protection scheme may take a long time to take effect. The effect is not obvious in the short term, and even the growth of the WECC index is even slower because it inhibits the economy. In the context of neglecting the environment and developing the economy at a high speed, the WECC in Scheme 1 has declined slowly, reaching only 0.324 in 2025.Compared with the other three schemes, the advantages of Scheme 3 are prominent and the upward trend is obvious. The WECC in this scheme will be in a “good” carrying state with a WECC index of 0.717 in 2025, indicating that coordinated development is the key to solve the WECC problem.

#### 3.1.3. Uncertainty Results

**Figure 6:**
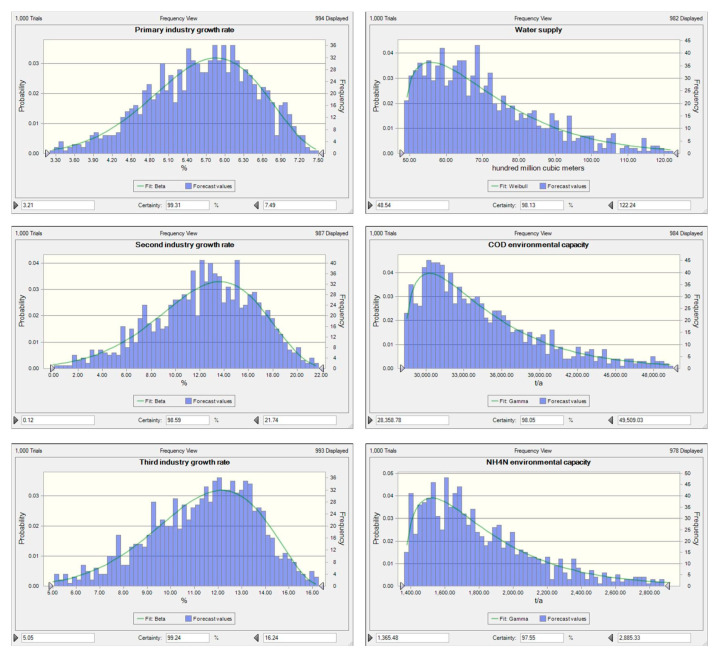
Probability distribution characteristics of six indicators. The probability of any interval within distribution range of each index can be obtained.

This study analyzed the uncertainty of the WECC results obtained in the economic and water environment by analyzing the real data of Fushun City in the past 20 years. Regression analysis was used to identify the relationship between the growth rate of the three industries and the growth rate of GDP. The probability distribution of the growth rate of the three industries was obtained in accordance with the Monte Carlo method (as shown in Figure 6). In terms of the water environment, regression analysis was used to identify the relationship between water availability, COD environmental capacity, and NH4N environmental capacity and precipitation. Then, the probability distribution of available water, COD environmental capacity, and NH_4_N environmental capacity were obtained in accordance with the Monte Carlo method (as shown in Figure 6).

This study considered the uncertainty of future social development and developed reasonable schemes based on past social development.

**Figure 7:**
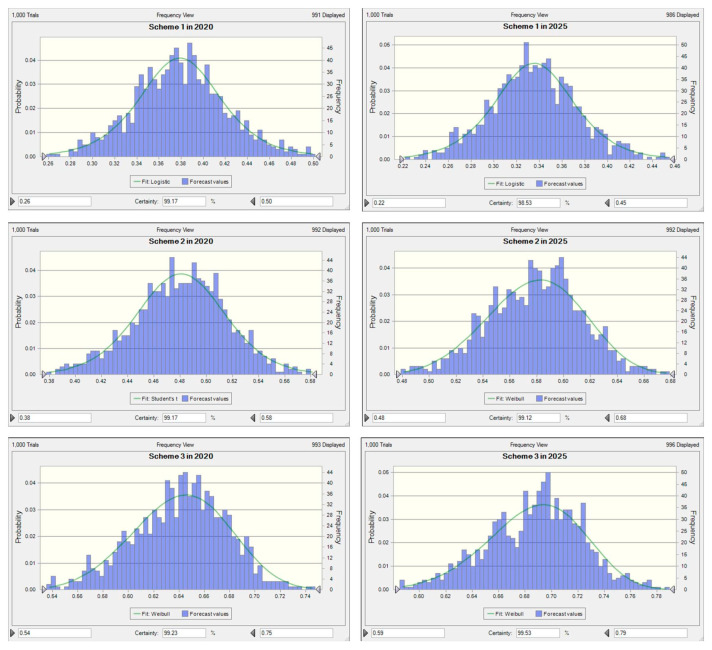
Probability distribution characteristics of the WECC indices of Scheme 1, Scheme 2, and Scheme 3 in 2020 and 2025.

The key parameters of the original scheme remained unchanged. From the perspective of the economy, Schemes 1–3 were divided in accordance with the actual probability distribution of the growth rates of the three industries in the past 20 years. The growth rates of the three industries in Scheme 1 were [6.18, 7.38]%, [14.97, 21.74]%, and [12.8, 16.24]%, and the realization probability was 30%. The growth rates of the three industries in Scheme 2 were [3.18, 5.25]%, [0.12, 10.53]%, and [5.05, 10.26]%, and the realization probability was 30%. The growth rates of the three industries in Scheme 3 were [5.25, 6.18]%, [10.53, 14.97]%, and [10.26, 12.8]%, and the realization probability was 40%. Table 6 shows the specific parameter changes. As shown in Figure 7, after the uncertainty analysis of the WECC results of the three schemes, the overall development trend of the WECC index of the three schemes did not change much. The WECC index of Scheme 1 continues to decline, to [0.22, 0.45] in 2025. The upward trend of the WECC index of Scheme 3 is still very clear, rising to [0.59, 0.79] in 2025. The WECC index of Scheme 2 is still not far off from the original scheme, and it can reach [0.48, 0.68] in 2025.

### 3.2. Discussion

The benchmark year (2015) in Fushun is in a poor carrying state. As shown in Figure 5, if the current situation continues to develop, the water environment in Fushun will not improve much by 2025. Therefore, this study proposes three schemes to improve the current poor water environment. In order to make the predictions of Schemes 1, 2, and 3 more convincing, we performed uncertainty analysis on the general results of WECC that we have obtained. From the perspective of uncertain results, this study has achieved its purpose. We have obtained the variation intervals of the WECC index values and the probability of each value in interval in each of the three schemes from 2016 to 2025. Compared with the WECC index value predicted when Zhang et al. (2014) studied and evaluated the development trend of water ecological carrying capacity in the Siping area by combining system dynamics and the analytic hierarchy process [28], the uncertain results obtained in this study will have more reference significance. Figure 7 shows only the WECC index values for the years 2020 and 2025. It is not difficult to see from the figure that the WECC index value of the maximum probability that can be achieved in Scheme 3 in 2025 is higher than that of the other two schemes, and the overall interval value is relatively high.

In addition, Scheme 3 has the highest probability of occurrence, reaching 40%. Therefore, Scheme 3 is the development path proposed in this study that can significantly improve the water environment in Fushun. This scheme does not emphasize the emphasis on a particular aspect of development but seeks a balance between the contradiction between economic development and water and environmental protection, and thus it controls the coordinated development of all indicators within a reasonable range. In terms of economy, the growth rates of the three industries decreased to [5.25, 6.18]%, [10.53, 14.97]% and [10.26, 12.8]%, respectively. In terms of population control, the urbanization level declined slightly to 0.65, and the migration coefficient of people in and out was 1.3 and 0.7, respectively. In terms of environmental protection, we have extensively applied emission reduction and pollution control technologies in industrial and agricultural production and raised the rates of COD and NH4N to 94% and 75%, respectively. Due to limited data, the water environment status assessment indicators selected in this study are not perfect, so the measures adopted in Scheme 3 can only be a general direction, and a lot of details and other measures should be further studied. This paper supplements the following suggestions on the basis of the research on the water environment carrying capacity in Fushun City: (1) actively adjust the industrial structure, vigorously promote the tertiary industry with low water consumption and waste water discharge, and adjust its growth rate to the same level as the secondary industry; (2) the old industrial areas in Fushun city are relatively concentrated, therefore, the establishment of larger sewage treatment plants can reduce the infrastructure investment and cost per unit of water treated, and also facilitate the improvement of the overall treatment technology level; (3) in order to effectively reduce the per capita sewage discharge capacity of Fushun city, the water price can be appropriately raised and the living sewage fee can be levied; (4) industry should pay more attention to the development of clean production processes and new technologies, especially water-saving technologies [29]. In agriculture, the development of organic agriculture can reduce the use of fertilizers and pesticides.

## 4. Conclusions

This study introduced the Monte Carlo method and SD model to establish a WECC evaluation index system and a WECC model for Fushun. Three schemes were designed to address ecological and environmental problems in the region, and the development trends of WECC in the region between 2016 and 2025 were predicted and evaluated using different schemes. The Monte Carlo classical sampling method was used to study the three schemes.

Results show that the rapid development of the economy adversely affects the water environment in Fushun, but reducing the pollutant emissions by sacrificing the economy will not effectively solve the ecological problems, it will only find a balance in the contradiction between economic development and water environmental protection. Through the adoption of a series of measures, including industrial restructuring, saving various resources, strengthening residents’ environmental awareness, and appropriately increasing environmental protection investment, the indicators are controlled within a reasonable range. In this manner, Fushun City can embark on the road of sustainable development and fundamentally improve the WECC.

An important contribution of this study is to propose a basis for making a scheme and a method to verify the feasibility of the scheme, namely the probability of whether the scheme can be realized. The system dynamics method is adopted to connect the scattered indexes into a whole, the Monte Carlo method is adopted to analyze the uncertainty of the panel data of each index, and then the probability of each index within a specific range is simulated. This probability is derived from the development of the existing society and is supported by real data, which has important reference significance for planning the future social development. Although the focus of this study is on the smooth water environment, the simulation methods adopted in this study can be used for reference for the simulation of water environment in other regions. It is suggested that big data should be used for management in future studies [30] to obtain more accurate and reasonable data to populate and execute the Monte Carlo method–system dynamics model.

## Figures and Tables

**Table 1:** Weights values of the evaluation indicators.

Object Hierarchy	Rule Hierarchy	Index Hierarchy	Weight Value
WECC in the Fushun area	System index for water resources	Primary industry water consumption	0.0305
		Second industry water consumption	0.0647
		Water supply	0.0484
		Water supply and consumption ratio	0.0355
	System index for water environment	COD environmental capacity	0.0637
		NH_4_N environmental capacity	0.0701
		Life COD production	0.0626
		Life NH_4_N production	0.0474
		COD emissions	0.0934
		NH_4_N emissions	0.0754
	System index for social economy	Total population	0.0752
		The level of urbanization	0.0723
		GDP	0.0940
		Primary industry growth rate	0.0366
		Second industry growth rate	0.0714
		Third industry growth rate	0.0588

**Table 2:** Equations.

Equation Number	Function of Equations	Equation	Supplementary Instruction
(1)	The function $\mu_{M}$ represents the membership of *M*	$\mu_{M}(x)=\{\begin{array}{cc} \frac{1}{m-x}x-\frac{l}{m-l} & x\in \left[l,m\right]\\ \frac{1}{m-u}x-\frac{u}{m-u} & x\in \left[m,u\right]\\ 0 & x\in \left(-\infty ,l\right]\cup^{\;}\left[u,+\infty \right) \end{array}$	*M* is a fuzzy number on the domain *R* (*R∈*[0, 1]); *l ≤ m ≤ u*; *l* and *u* represent the lower and upper bounds of *M*; *m* is the median of the membership degree of *M*; The general triangular fuzzy number *M* is expressed as (*l, m, u*).
(2)	$D_{i}^{k}$ is the comprehensive fuzzy value of the element *i* of the *K*th layer (initial fuzzy value)	$D_{i}^{k}=\sum_{j=1}^{n}a_{ij}^{k}/\left(\sum_{i=1}^{n}\sum_{j=1}^{n}a_{ij}^{k}\right),i=1,2,\ldots ,n$	
(3)	The probability of $M_{1}\geq M_{2}$ (It is defined by the triangular fuzzy function)	$v\left(M_{1}\geq M_{2}\right)=sup_{x\geq y}\left[min\left(\mu_{M_{1}}\left(x\right),\mu_{M_{2}}\left(y\right)\right)\right]$ *or* $v\left(M_{1}\geq M_{2}\right)=\{\begin{array}{cc} 1 & m_{1}\geq m_{2}\\ \frac{l_{2}-u_{1}}{\left(m_{1}-u_{1}\right)-\left(m_{2}-l_{2}\right)} & m_{1}\leq m_{2},u_{1}\geq l_{2}\\ 0 & otherwise \end{array}$	*M*_1_(*l*_1_,*m*_1_,*u*_1_) and *M*_2_(*l*_2_,*m*_2_,*u*_2_) are any given two fuzzy numbers
(4)	Definition of the probability that one fuzzy number is greater than the other *K* fuzzy numbers	$v\left(M\geq M_{1},M_{2},\ldots \ldots ,M_{K}\right)=min\left(M\geq M_{i}\right),i=1,2,\ldots ,k$	
(5)	For the indicator, “the smaller the value, the more favorable the WECC”	$Q_{ij}=\frac{\underset{j=1,2,\ldots ,m}{\max}e_{ij}-e_{ij}}{\underset{j=1,2,\ldots ,m}{\max}e_{ij}-\underset{j=1,2,\ldots ,m}{\min}e_{ij}}$	$Q_{ij}$ is the score of *i*-index (*i* = 1, 2, ..., 15, 16) in *j*-scheme (*j* = 1, 2, 3, 4); $e_{ij}$ is the value of *i*-index in *j*-scheme; $e_{ij}^{*}$ is the optimized value of interval index, which is the average of $e_{ij}$ in the study.
(6)	For the indicator, “the larger the value, the more favorable the WECC”	$Q_{ij}=\frac{e_{ij}-\underset{j=1,2,\ldots ,m}{\min}e_{ij}}{\underset{j=1,2,\ldots ,m}{\max}e_{ij}-\underset{j=1,2,\ldots ,m}{\min}e_{ij}}$	
(7)	For the indicator, “the closer the value is to a certain value, the more favorable the WECC”	$Q_{ij}=1-\frac{\left|e_{ij}-e_{ij}^{*}\right|}{\underset{j=1,2,\ldots ,m}{\max}e_{ij}-\underset{j=1,2,\ldots ,m}{\min}e_{ij}}$	
(8)	WECC index $R_{j}$ calculation formula	$R_{j}=\sum_{i=1}^{n}C_{i}Q_{ij}$	$Q_{ij}$ is the standardized score of *i*-index in *j*-scheme; $C_{i}$ is the weight of *i*-index determined by FAHP.

**Table 3:** Error test.

Index		2006	2007	2008	2009	2010	2011	2012	2013	2014	2015
GDP (hundred million Yuan)	Actual value	457.8	547.2	662.4	698.6	890.2	1113.3	1242.4	1340.4	1276.6	1216.5
	Simulation value	501.5	586.3	624	685.5	851.1	1081	1206	1316	1289	1230
	Relative error	−9.55%	−7.15%	5.80%	1.88%	4.39%	2.90%	2.93%	1.82%	−0.97%	−1.11%
Total population (million people)	Actual value	2.24	2.24	2.23	2.23	2.21	2.20	2.19	2.18	2.17	2.16
	Simulation value	2.24	2.23	2.23	2.22	2.21	2.20	2.19	2.18	2.17	2.15
	Relative error	0.00%	−0.32%	−0.31%	−0.50%	−0.14%	−0.23%	−0.25%	−0.07%	−0.28%	−0.19%
Urban population (ten thousand people)	Actual value	148.32	147.96	147.30	146.41	145.48	144.54	143.64	142.32	142.60	141.20
	Simulation value	148.30	147.50	146.80	145.70	145.30	144.20	143.20	142.20	141.10	142.30
	Relative error	−0.01%	−0.31%	−0.34%	−0.48%	−0.13%	−0.24%	−0.31%	−0.08%	−1.05%	0.78%
NH_4_N production(t)	Actual value	6688	6731	6743	6762	6863	7003	7054	7083	7125	7148
	Simulation value	6625	6711	6766	6801	6921	7040	7105	7162	7111	7023
	Relative error	−0.94%	−0.30%	0.34%	0.58%	0.85%	0.53%	0.72%	1.12%	−0.20%	−1.75%
COD production(t)	Actual value	163,575	163,732	163,744	164,318	164,796	164,880	164,730	164,556	164,002	163,050
	Simulation value	161,678	161,821	162,164	163,201	163,814	164,800	165,581	166,746	164,846	161,684
	Relative error	−1.16%	−1.17%	−0.96%	−0.68%	−0.60%	−0.05%	0.52%	1.33%	0.51%	−0.84%
Total water consumption (One hundred million cubic meters)	Actual value	5.14	5.056	4.608	4.923	4.924	4.74	4.43	4.18	4.18	4.85
	Simulation value	4.94	5.29	4.57	4.9	4.88	4.78	4.66	4.49	4.19	4.69
	Relative error	−3.89%	4.63%	−0.82%	−0.47%	−0.89%	0.84%	5.19%	7.42%	0.24%	−3.30%

**Table 4:** Water environment carrying capacity (WECC) carrying state table.

WECC Index	Carrying State
0.8–1.0	excellent
0.6–0.8	good
0.4–0.6	general
0.2–0.4	poor
0–0.2	very poor

**Table 5:** Improvement schemes of water environment carrying capacity in Fushun area.

Scheme	Key Development Direction	Detailed Procedures
Original scheme	Maintained the existing development model of Fushun area	The urbanization rate remained at 0.69, with small natural and mechanical changes of population; The government lays emphasis on economic development, industrial investment and agricultural investment remained at the average level in the past five years, with the growth rate of the three industries at 6.12%, 14.71%, and 12.67%, respectively; The current COD and NH_4_N treatment rates remained at 92% and 70% in terms of environmental treatment.
Scheme 1	Economic development, especially industrial progress, is placed in the first place in social development	The urbanization level is stable at 0.8, and the coefficient of migration in and out is 1.5 and 0.5 respectively; The government strongly supported industrial development, and the coefficient of industrial policy reached 1.45. The investment in industry was overwhelming that in agriculture and tertiary industry. The growth rate of the three industries rose to 6.69%, 18.77% and 14.67%; The environmental protection level do not significantly improve in comparison with the original scheme.
Scheme 2	Economic development was restrained to reduce the emission pollutants	Industry, as the main driver of water environmental pollution, was restricted, and the coefficient of industrial policy dropped to 0.9.The overall economic development will be very slow, the growth rate of the three major industries will drop to 4.04%, 4.35% and 6.87% respectively, and the urbanization level will also drop to 0.67.; With the implementation of government regulations and policies on environmental protection, the COD and NH_4_N treatment rates have increased to 96% and 80%.
Scheme 3	Enhance environmental protection while comprehensively developing the economy	Emission reduction and pollution control technology were widely promoted in industrial and agricultural production. The treatment rates of COD and NH_4_N increased to 94% and 75%, respectively, compared with the original scheme. In terms of economy, the growth rate of the three industries (5.46%, 12.16%, and 10.71%) decreased slightly. The GDP growth rate slowed down, and urbanization level slightly decreased to 0.65, with the coefficient of migration in and out is 1.3 and 0.7 respectively.

Note: the key parameters controlling important evaluation indicators in the scheme setting of this study are mainly migration coefficient, migration coefficient, urbanization rate, growth rate of primary industry, growth rate of secondary industry, growth rate of tertiary industry, COD treatment rate and NH4N treatment rate.

**Table 6:** Parameter settings for four scenarios after uncertainty analysis.

Index	Original Scheme	Scheme 1	Scheme 2	Scheme 3
Immigration coefficient	1	1.5	0.5	0.7
Emigration coefficient	1	0.5	1.5	1.3
The level of urbanization	0.69	0.8	0.67	0.65
Primary industry growth rate (%)	6.18	[6.18, 7.38]	[3.18, 5.25]	[5.25, 6.18]
Second industry growth rate (%)	14.97	[14.97, 21.74]	[0.12, 10.53]	[10.53, 14.97]
Third industry growth rate (%)	12.8	[12.8, 16.24]	[5.05, 10.26]	[10.26, 12.8]
COD processing rate (%)	92	92	96	94
NH_4_N processing rate (%)	70	70	80	75
